# EpCAM-Binding DARPins for Targeted Photodynamic Therapy of Ovarian Cancer

**DOI:** 10.3390/cancers12071762

**Published:** 2020-07-02

**Authors:** Dirk van den Brand, Sanne A. M. van Lith, Jelske M. de Jong, Mark A. J. Gorris, Valentina Palacio-Castañeda, Stijn T. Couwenbergh, Mark R. G. Goldman, Inge Ebisch, Leon F. Massuger, William P. J. Leenders, Roland Brock, Wouter P. R. Verdurmen

**Affiliations:** 1 Department of Biochemistry, Radboud Institute for Molecular Life Sciences (RIMLS), Radboud University Medical Center, Geert Grooteplein 28, 6525 GA Nijmegen, The Netherlands; Dirk.vandenBrand@radboudumc.nl (D.v.d.B.); jelske.marije@me.com (J.M.d.J.); Valentina.Palacio-Castaneda@radboudumc.nl (V.P.-C.); Stijn.Couwenbergh@radboudumc.nl (S.T.C.); info@markgoldman.nl (M.R.G.G.); William.Leenders@radboudumc.nl (W.P.J.L.); Roland.Brock@radboudumc.nl (R.B.); 2 Department of Obstetrics and Gynaecology, Radboud University Medical Center, Geert Grooteplein 10, 6525 GA Nijmegen, The Netherlands; Leon.Massuger@radboudumc.nl; 3 Department of Radiology and Nuclear Medicine, Radboud University Medical Center, Geert Grooteplein 10, 6525 GA Nijmegen, The Netherlands; Sanne.vanLith@radboudumc.nl; 4 Department of Tumor Immunology, Radboud Institute for Molecular Life Sciences (RIMLS), Radboud University Medical Center, Geert Grooteplein 10, 6525 GA Nijmegen, The Netherlands; Mark.Gorris@radboudumc.nl; 5 Department of Obstetrics and Gynaecology, Canisius Wilhelmina Hospital, Weg door Jonkerbos 100, 6532 SZ Nijmegen, The Netherlands; I.Ebisch@cwz.nl

**Keywords:** designed ankyrin repeat protein, epithelial cell adhesion molecule, ovarian cancer, targeted photodynamic therapy

## Abstract

Ovarian cancer is the most lethal gynecological malignancy due to late detection associated with dissemination throughout the abdominal cavity. Targeted photodynamic therapy (tPDT) aimed at epithelial cell adhesion molecule (EpCAM), overexpressed in over 90% of ovarian cancer metastatic lesions, is a promising novel therapeutic modality. Here, we tested the specificity and activity of conjugates of EpCAM-directed designed ankyrin repeat proteins (DARPins) with the photosensitizer IRDye 700DX in in vitro and in vivo ovarian cancer models. EpCAM-binding DARPins (Ec1: K_d_ = 68 pM; Ac2: K_d_ = 130 nM) and a control DARPin were site-specifically functionalized with fluorophores or IRDye 700DX. Conjugation of anti-EpCAM DARPins with fluorophores maintained EpCAM-specific binding in cell lines and patient-derived ovarian cancer explants. Penetration of DARPin Ec1 into tumor spheroids was slower than that of Ac2, indicative of a binding site barrier effect for Ec1. DARPin-IRDye 700DX conjugates killed EpCAM-expressing cells in a highly specific and illumination-dependent fashion in 2D and 3D cultures. Furthermore, they effectively homed to EpCAM-expressing subcutaneous OV90 xenografts in mice. In conclusion, the high activity and specificity observed in preclinical ovarian cancer models, combined with a high specificity in patient material, warrant a further investigation of EpCAM-targeted PDT for ovarian cancer.

## 1. Introduction

Ovarian cancer is the most lethal gynecological malignancy in the developed world and the sixth most frequent cause of cancer-related deaths worldwide [1]. At first diagnosis, these tumors have often already metastasized throughout the abdominal cavity [2]. The current treatment strategy is maximal cytoreductive surgery followed by chemotherapy [3]. However, median progression-free survival (PFS) is only 18 months and tumors almost always develop chemotherapy resistance [4]. Intraperitoneal (IP) (hyperthermic) chemotherapy, angiogenesis inhibitors or poly(ADP-ribose) polymerase (PARP) inhibitors increase PFS, but have no or little effect on overall survival [5,6,7,8,9,10]. Hence, there remains an urgent need for novel therapeutic options for disseminated ovarian cancer. 

Photodynamic therapy (PDT) is a therapeutic modality in which a photosensitizer (PS) is activated into a short-lived excited state by photons of a specific wavelength [11]. During return to its ground state, energy is transferred to biomolecules or oxygen, yielding reactive oxygen species (ROS) that induce peroxidation of membrane lipids and instantaneous cell death [12]. Various PSs are approved by the FDA [13], and some of these have been investigated (pre)clinically for the treatment of disseminated ovarian cancer. In clinical trials, PDT for peritoneal metastases of ovarian cancer has been shown to prolongate disease-free survival. However, a lack of tumor specificity of the PSs led to significant skin toxicity and other side effects such as bowel perforation, capillary leak syndrome and anastomotic leakages [14,15,16,17,18]. Targeted delivery of a PS to tumor cells, an approach called targeted photodynamic therapy (tPDT), can be achieved by conjugation to tumor-targeting moieties (e.g., peptides, monoclonal antibodies) or by encapsulating the PS in tumor-targeting nanoparticles [18,19,20]. The concept of tPDT is that by concentrating PSs to a tumor, efficacy at the target cells is improved, whilst toxicity of non-target cells is diminished.

Epithelial cell adhesion molecule (EpCAM) is a transmembrane glycoprotein that is involved in cell adhesion, signaling, migration, proliferation and differentiation [21,22]. More than 90% of metastatic lesions of malignant ovarian tumors, as well as many other carcinomas, overexpress EpCAM relative to normal epithelial cells and normal mesothelium [21,23,24,25]. Furthermore, EpCAM expression in tumor cells is not confined to the basolateral side as in normal cells, making cancer cells better accessible to EpCAM-targeting agents via the apical side [26]. EpCAM is therefore a suitable target for tPDT of ovarian cancer cells. Thus far, tPDT for metastasized ovarian cancer has been performed with PSs conjugated to folic acid, targeting the folate receptor [27,28], or various full-length IgG molecules [29,30,31,32,33]. For IgG, however, the long plasma half-life requires an extended time between injection and illumination to yield sufficient systemic clearance, which is impractical and raises the risk for off-target effects [34]. In parallel, IgGs have poor tumor penetration due to their large size [35,36]. Low molecular weight non-antibody formats, such as designed ankyrin repeat proteins (DARPins), represent attractive alternatives that overcome these disadvantages [37,38,39,40,41]. Various DARPins have already shown good safety and efficacy profiles in clinical studies, indicating potential for clinical translation [42]. Here, we investigated the feasibility of tPDT of ovarian cancer using previously described anti-EpCAM DARPins [43], conjugated to the photosensitizer IRDye 700DX. Using the EpCAM-binding DARPins Ec1 and Ac2, we observed a high activity and specificity in 2D and 3D in vitro ovarian cancer models, a high specificity in patient material and an effective tumor homing in vivo, illustrating the potential of EpCAM-targeted PDT using DARPins. 

## 2. Results

### 2.1. Production of DARPin-Conjugates

**Figure 1:**
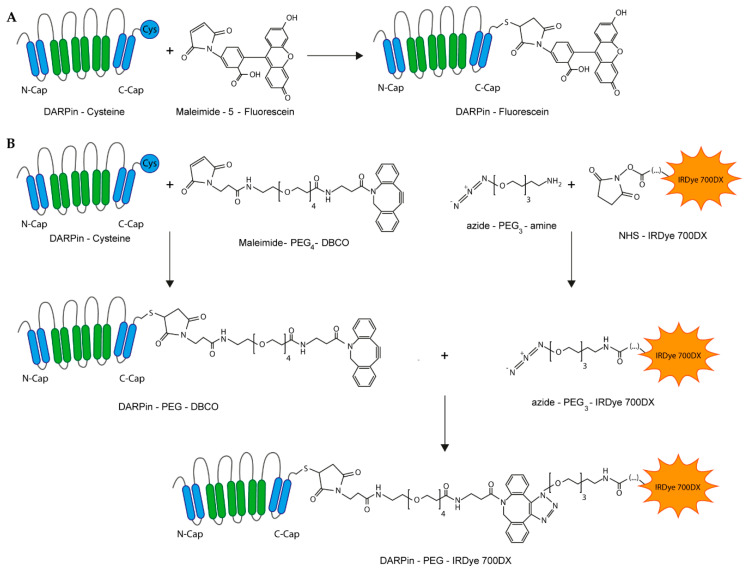
Schematic representation of DARPin conjugations. (**A**) Synthesis of DARPin-fluorescein conjugates. The same strategy was employed for coupling Alexa Fluor 680. (**B**) Three-step preparation of DARPin-IRDye 700DX conjugates.

For this study, the DARPins Ec1 and Ac2 were utilized which exhibit a high binding affinity to distinct epitopes on EpCAM with dissociation constants of 68 pM and 130 nM, respectively [43]. DARPins were successfully expressed and purified by IMAC (Figure S1). To visualize EpCAM binding, DARPins were coupled to fluorescein or Alexa Fluor 680 using maleimide-thiol chemistry (Figure 1A). DARPin-PS conjugates were prepared by first coupling a maleimide-PEG4-DBCO heterobifunctional linker to a carboxyterminal cysteine (Figure 1B). As IRDye 700DX was only available as an NHS ester, we introduced an azide functionality to enable regioselective coupling to the DBCO-functionalized DARPins. First, the IRDye 700DX with NHS ester was coupled to an amine-PEG3-azide in organic solvent at high yield (>85%) and then coupled via copper-free click chemistry to the DBCO-functionalized DARPin, yielding DARPin-IRDye 700DX constructs (Figure 1B). All DARPin-IRDye 700DX conjugates induced production of singlet oxygen with equal efficiency upon illumination with 690 nm light and at similar levels as the parent compound IRDye 700DX, as illustrated by bleaching of the singlet oxygen generation reporter molecules p-nitrosodimethylaniline (RNO) and 9,10-anthracenediylbis(methylene) malonic acid (ABDA) (Figure S2).

### 2.2. EpCAM Binding in 2D Cell Cultures

**Figure 2:**
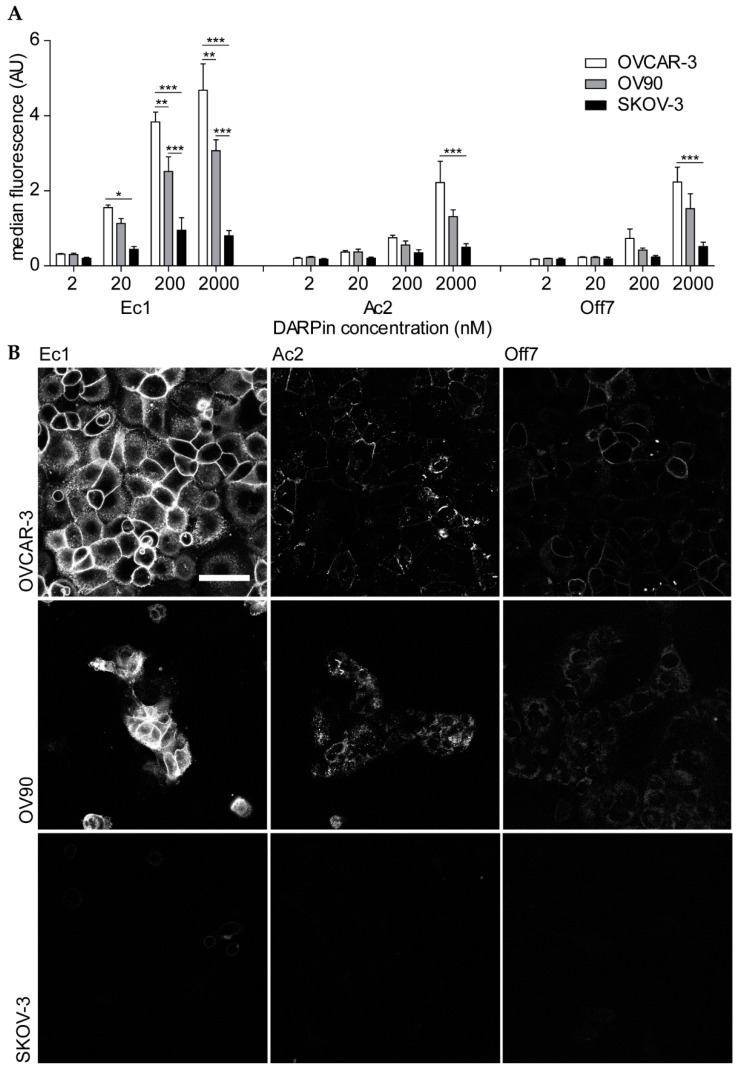
Binding of DARPin-fluorescein conjugates to ovarian cancer cell lines. (**A**) Flow cytometry of cells that were incubated with different concentrations of DARPins. Mean ± s.e.m. is shown. A two-way ANOVA with Bonferroni post hoc test was used to calculate statistical differences. * *p* < 0.05, ** *p* < 0.01, *** *p* < 0.001. OVCAR-3 *n* = 5; OV90, SKOV-3 *n* = 3. (**B**) Confocal microscopy images of cells that were incubated with 200 nM DARPin-fluorescein conjugates. Laser power and gain were kept constant and brightness and contrast settings were adjusted equally. Scale bar represents 50 µm; *n* = 3; data from a representative experiment are shown.

The ovarian cancer cell lines OVCAR-3, OV90 and SKOV-3 were selected to evaluate the EpCAM-binding characteristics of Ec1 and Ac2 conjugates. As observed by flow cytometry, OVCAR-3 and OV90 cell lines showed high EpCAM expression and SKOV-3 showed low EpCAM expression (Figure S3). DARPin binding was concentration dependent and Ec1 showed higher binding than Ac2 (Figure 2A; for representative flow cytometry histograms, see Figure S4). This is in line with a previous report [43], indicating that the site-specific conjugation of a fluorophore did not affect EpCAM binding and the higher affinity of Ec1 in comparison to Ac2 was maintained. 

To visualize cellular binding and uptake of the DARPins into cells, we performed confocal microscopy of all cells after incubation with 200 nM of the constructs. On OVCAR-3 and OV90 cells, we observed mainly membrane staining, which was more prominent for Ec1 than for Ac2, and nearly absent for Off7. With the same settings, little fluorescence was observed for SKOV-3 cells (Figure 2B).

### 2.3. Phototoxicity of DARPin-IRDye 700DX Conjugates in 2D and 3D Cell Cultures

**Figure 3:**
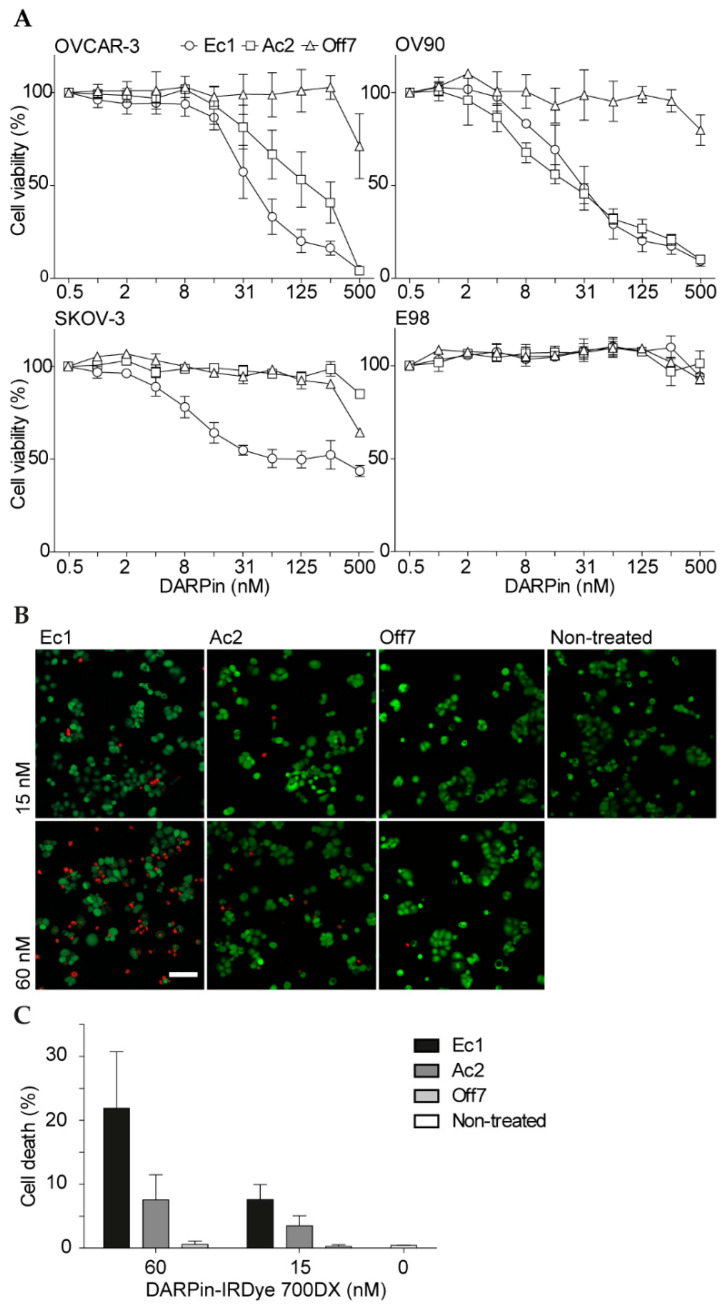
Cell viability after DARPin-IRDye 700DX incubation and illumination. (**A**) Cell viability in monolayers of OVCAR-3, OV90, SKOV-3 and E98 cells. Cells were incubated with different concentrations of DARPin-IRDye 700DX conjugates for 2 h followed by illumination (60 J/cm^2^ at 100 mW/cm^2^). After overnight incubation, cell viability was determined with a resazurin cell viability assay. Mean and s.e.m. are shown; *n* = 3 for OV90 and OVCAR-3 cells, *n* = 2 for SKOV-3 and E98 cells. The scale on the *x*-axis is logarithmic. (**B**) Confocal microscopy imaging of cell viability in OVCAR-3 cells after 30 min incubation with 15 or 60 nM DARPin-IRDye 700DX conjugates and illumination (60 J/cm^2^ at 100 mW/cm^2^). Representative microscopy images of cell death after PDT are shown. Red indicates propidium iodide, green indicates calcein. Scale bar represents 100 µm. (**C**) Quantification of cell death using a macro in ImageJ. For each experiment, 12–20 images were analyzed per condition; mean is shown; error bar represents the range of independently performed experiments (*n* = 2).

The activity of DARPin-IRDye 700DX conjugates was assessed towards OVCAR-3, OV90, SKOV-3 and EpCAM-negative E98 astrocytoma cells. OVCAR-3 and OV90 were the most sensitive to EpCAM-targeted photodynamic therapy (Figure 3A), consistent with the high EpCAM expression levels on these cells (Figure S3). Interestingly, Ec1 and Ac2 had comparable IC_50_ values on OV90 cells (Ec1: 33 nM [95% CI: 26–42 nM]; Ac2: 28 nM [95% CI: 21–36 nM]), while on OVCAR-3 cells the Ec1 conjugate was more potent than the Ac2 conjugate (IC_50_ Ec1: 43 nM [95% CI: 34–54 nM]; IC_50_ Ac2: 128 nM [95% CI: 95–173 nM]). On SKOV-3 cells, only Ec1 showed an effect, although even 500 nM of Ec1 conjugate did not induce cell death of all cells. This observation suggests that the higher binding affinity of Ec1 is especially useful in cells that have intermediate or low EpCAM expression. Overall, differences between phototoxicity of Ec1- and Ac2-conjugates were smaller when compared to differences in binding as observed in flow cytometry. For Off7, although at higher concentrations cell-associated fluorescence was similar to the one of Ac2 (Figure 2), no toxicity was observed, possibly due to a better binding at lower concentrations of Ac2 or a stronger membrane localization of Ac2 as compared to Off7, which is known to affect the therapeutic activity [37]. No significant toxicity was observed in EpCAM-negative E98 astrocytoma cells. To characterize the illumination-dependency of the cytotoxic effect, we incubated OVCAR-3 cells with different concentrations of the DARPin-IRDye 700DX conjugates and then exposed the cells to various light dose rates as well as increasing total light doses. For Ec1-IRDye 700DX and Off7-IRDye 700DX, no dose (rate) dependency was observed, since Ec1-IRDye 700DX was equally potent and Off7-IRDye 700DX did not induce phototoxic effects in all conditions tested. For Ac2-IRDye 700DX, however, we noted that both a lower light dose and light dose rate reduced efficacy, while increasing light dose or dose rate had no additional effect (Figure S5). To investigate whether the phototoxic effects are ROS-mediated, we tested the effects of histidine and sodium azide as ROS scavengers on the phototoxicity of 50 nM Ec1-IRDye 700DX. However, scavengers did not affect toxicity in these conditions (Figure S6).

To visualize the phototoxicity mediated by tPDT, OVCAR-3 cells were treated with DARPin-IRDye 700DX conjugates, stained with calcein AM (to identify live cells) and PI (to identify late apoptotic and necrotic cells) and imaged with confocal microscopy (Figure 3B). The short assay duration implies that PI-positivity reflects necrosis and not (late) apoptosis caused by PDT-mediated acute and extensive membrane damage [11]. An increased number of necrotic cells was observed with the higher DARPin-IRDye 700DX concentration, with 22% necrotic cells when using 60 nM Ec1-IRDye 700DX (Figure 3C). In comparison, both 60 nM Ac2-IRDye 700DX and 15 nM Ec1-IRDye 700DX resulted in 8% acute cell death, with virtually no acute toxicity observed for the Off7-IRDye 700DX conjugates. Since the percentages of cells undergoing acute cell death are lower than the overall reduction in viability after an overnight incubation at similar concentration (cf. Figure 3A), the co-occurrence of distinct mechanisms (i.e., necrosis and apoptosis) can be assumed.

### 2.4. Penetration and Activity in Spheroids

**Figure 4:**
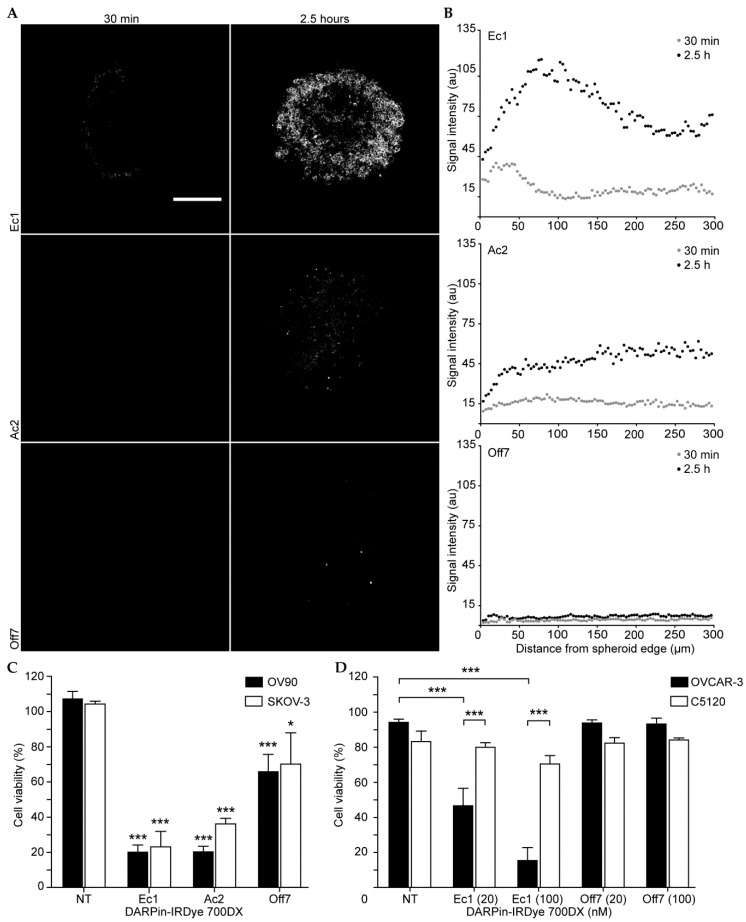
DARPin penetration and phototoxicity in 3D culture models. (**A**) DARPin uptake in a spheroid model of OV90 cells. DARPins were incubated for 30 min or 2.5 h and imaged with confocal microscopy after a SeeDB clearing protocol to enable visualization of fluorescence throughout the spheroid. Z-stacks are provided in Figure S7. The scale bar denotes 200 µm. (**B**) Quantification of the signal intensity from DARPin uptake in spheroids. Each data point represents the measurement of the average signal intensity at the indicated distance from the spheroid rim. For each condition, a representative spheroid was used for quantitative analysis. (**C**) Spheroids from both OV90 and SKOV-3 cells were cultured and subsequently incubated with 500 nM of the different DARPin-IRDye 700DX conjugates followed by washing and illumination (60 J/cm^2^ at 100 mW/cm^2^). Cell viability was assessed with the APH assay and all data were normalized to non-treated, illuminated samples. Mean and s.e.m. are shown (*n* = 3). (**D**) Analysis of cell viability of OVCAR-3/C5120 co-cultures in Matrigel. After 2 h of incubation with DARPin-IRDye 700DX conjugates, the co-cultures were illuminated and cell viability was assessed by the addition of propidium iodide. The percentage cell viability was calculated by dividing the number of dead cells of each cell line by the total number of cells of the same cell line. Mean and s.e.m. are shown (*n* = 3). Statistical significance was determined by comparing mean signal intensity with non-treated samples. In addition, the mean signal intensity between different cell lines treated with the same construct and concentration were compared. A two-way ANOVA with Bonferroni post hoc test was employed for statistical analysis. * *p* < 0.05, *** *p* < 0.001.

To assess the tumor-penetrating ability of DARPins, a 3D tumor spheroid model of OV90 cells was used. After 30 min incubation, Ec1 could only be detected on the outer rim of the spheroid (Figure 4A and Figure S7), as also evident from its radial intensity profile (Figure 4B). Importantly, due to the clearing protocol, these intensity profiles are free from bias due to incomplete penetration of light (Figure S7) [44]. After 2.5 h of incubation, Ec1 showed a full penetration. Ac2, by comparison, reached complete penetration already after 30 min, though intensity increased further until 2.5 h of incubation. This indicates that the low affinity DARPin is less hindered by a binding site barrier, presumably because it exhibits faster off rates. No penetration was observed for Off7.

To evaluate PDT in 3D, spheroids were incubated with 500 nM of Ec1-, Ac2-, or Off7-IRDye 700DX conjugates for 2 h. After washing, the spheroids were illuminated (60 J/cm^2^ at 100 mW/cm^2^) and an APH assay was performed the subsequent day. The APH assay, which is based on quantifying cytosolic acid phosphatase activity, is a particularly reliable method to determine cell viability in spheroids, as it does not require spheroid dissociation, provides linear results and is highly sensitive [45]. Cell viability in treated OV90 spheroids decreased equally for Ec1- and Ac2-IRDye 700DX-treated spheroids, leading to mean cell viabilities of 20 ± 4.0% (Figure 4C). Surprisingly, in EpCAM-low SKOV-3 spheroids, an almost equal efficiency was observed, with Ec1- and Ac2 both effectively decreasing cell viability to a mean cell viability of 23 ± 8.8% and 36 ± 3.2%, respectively (Figure 4C). 

To evaluate the specificity of targeting in a 3D environment, PDT with DARPin-IRDye 700DX conjugates was evaluated in a 3D co-culture model of cytoplasmically stained OVCAR-3 and C5120 fibroblasts (with low EpCAM expression [46]), in Matrigel. Co-cultures were imaged with confocal microscopy (Figure S8), and cell viability was quantified for each cell type (Figure 4D). Cell viability of both cell lines was unaffected with illumination only or when treated with Off7-IRDye 700DX. At 20 and 100 nM, Ec1 conjugates significantly decreased cell viability of OVCAR-3 cells to 47 ± 10% and 15 ± 7.4%, respectively. A statistically non-significant decrease in cell viability from 83 ± 5.9% (non-treated) to 70 ± 4.7% was observed for the C5120 cells at 100 nM Ec1-IRDye 700DX.

### 2.5. Tumor Targeting and Activity in OV90 Subcutaneous Xenografts

**Figure 5:**
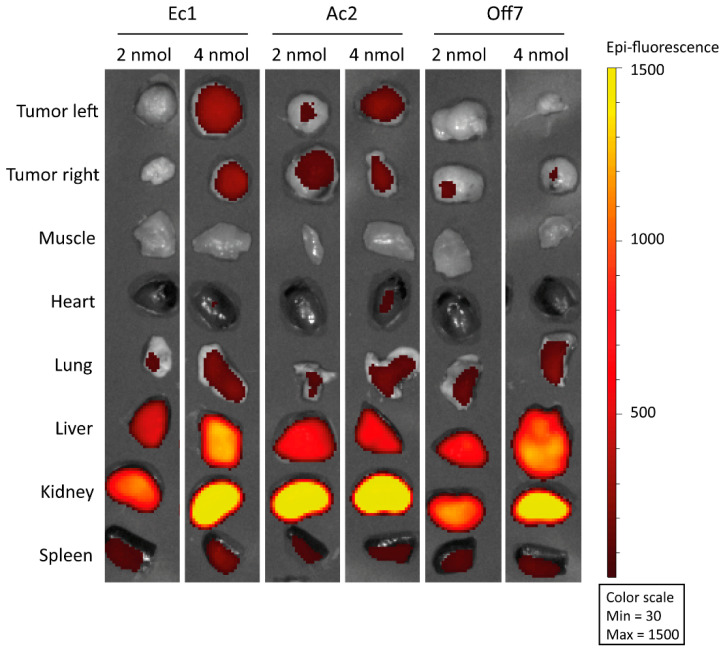
Visualization of IRDye 700DX-derived fluorescence in OV90 tumors and organs harvested from mice at 4 h after intravenous injection with either 2 or 4 nmol of the various DARPin-IRDye 700DX conjugates.

To assess the tumor-targeting capacity of the DARPins in vivo, we visualized fluorescent signal derived from the IRDye 700DX in the tumor and organs at 4 h after intravenous injection of either 2 or 4 nmol of the constructs into mice bearing OV90 subcutaneous xenografts (Figure 5). For both Ec1 and Ac2, tumor uptake was higher at 4 nmol than at 2 nmol, while hardly any signal was detected for Off7. No uptake was found in muscle or heart tissue, while renal uptake was very high. Furthermore, some targeting to the lung, liver and spleen was observed. Though tumor targeting was observed by fluorescence imaging, injection of 4 nmol of the conjugates and illumination with 150 J/cm^2^ 690 nm light did not lead to changes in tissue morphology of the tumor, cleaved caspase-3 or γH2A.X expression at 24 h after illumination (150 J/cm^2^ at 170 mW/cm^2^), when compared to the non-illuminated tumor on the other flank (Figure S9). Notably, there were also no signs of aspecific phototoxicity or acute burning of tissue. 

### 2.6. Cell Specificity in Patient Samples

**Figure 6:**
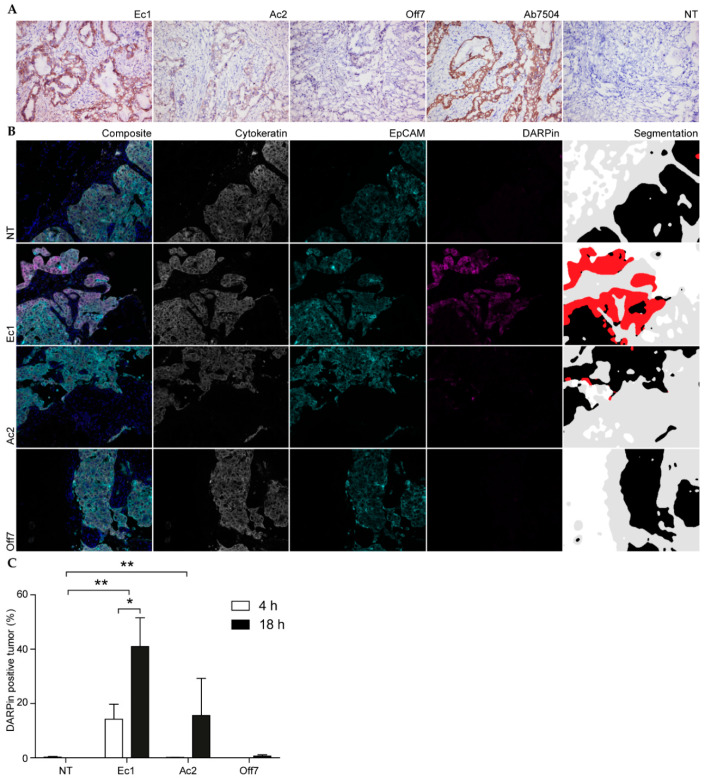
Binding of DARPins to primary tumor tissue. (**A**) Cryosections of ovarian cancer tissue incubated with DARPins and visualized with DAB immunohistochemistry. (**B**) Representative images of metastatic ovarian tumor tissue that was incubated alive ex vivo before fixation. Slices were incubated for 4 h with DARPins. They were subsequently processed and stained via multiplex immunohistochemistry. A composite image is shown with blue-stained nuclei. This is followed by the separate image of each staining. Lastly, a segmentation image is added that gives a clear overview of the presence of stroma (gray), tumor (black) and DARPin (red). The DARPin-positive areas in these experiments were also EpCAM positive, but are only shown in red. (**C**) The percentage of positive tumor area after 4 and 18 h was calculated by dividing the DARPin-positive tumor surface area by the total tumor surface area (sum of DARPin-positive and negative tumor surface areas). Mean and s.e.m. are shown. Significance was determined with a two-way ANOVA and Bonferroni post hoc test. * *p* < 0.05, ** *p* < 0.01. *n* = 3.

Co-cultures in Matrigel still provide a highly simplified model of a complex tumor environment. To investigate the tumor cell specificity of the EpCAM-targeting DARPins in human samples, frozen tissue sections of human serous papillary ovarium carcinoma were stained with an anti-EpCAM antibody. The staining patterns of both Ec1 and Ac2 showed specific staining of EpCAM-positive cells (Figure 6A). The cell-staining pattern coincided with the pattern shown by antibody immunohistochemistry. Both EpCAM-targeting DARPins did not bind to other cells in the tissue, and the control DARPin Off7 did not show any binding at all, indicating that cell specificity is maintained in the natural context in primary patient-derived tumors.

Finally, to learn about binding and penetration in a viable ex vivo 3D tumor environment, DARPins were incubated on sections derived from metastatic ovarian tumor tissue of four patients with stage IIIC epithelial ovarian cancer. This tissue was collected from patients that underwent cytoreductive debulking surgery for ovarian cancer. The tumor deposits were incubated with DARPins for 4 and 18 h and processed for multiplex immunohistochemistry. Cytokeratin was stained in order to identify all cells of epithelial origin. Furthermore, EpCAM and DARPins were stained using anti-EpCAM and anti-FLAG antibodies, respectively, in order to visualize cell specificity.

Despite its origin, one of the tumor samples was EpCAM negative and was therefore not included in the analysis. Ec1 showed a prominent tumor binding after 4 h (Figure 6B), whereas after 18 h specific binding was evident for both EpCAM-targeting DARPins. In contrast, the non-specific binding of Off7 remained low. Quantification of the signals shows that the area percentage that is both EpCAM positive and stained with Ec1 increased from 14% after 4 h to 41% after 18 h. Ac2 binding was not evident after 4 h (Figure 6B,C), even though the percentage area overlap with the anti-EpCAM stain increased to 16% after 18 h of incubation. This was considerably higher than the low degree of non-specific binding of Off7 that was observed after 18 h (0.7%). For Ec1, the penetration into the sample core was lower than the penetration on the edge of the sample (Figure 6B). Overall, DARPin binding was higher for Ec1 than for Ac2.

## 3. Discussion

In this study, we showed the feasibility and specific phototoxic activity of IRDye 700DX coupled to EpCAM-targeting DARPins for targeted PDT in ovarian cancer. Both EpCAM-targeting DARPins, Ec1 and Ac2, showed binding and the ability to effectively induce cell death in both 2D and 3D models of epithelial ovarian cancer. Binding efficiency correlated with receptor expression and dissociation constant as expected. Cells with higher EpCAM expression were more vulnerable for EpCAM-targeted PDT.

Ec1 showed the highest binding to EpCAM, consistent with earlier research [43]. In line with these results, the highest cell killing activity in a monolayer model was also observed for Ec1. At the highest concentrations tested, we would expect that 100% of the epitopes are occupied with either EpCAM-targeting DARPin. However, even in saturation, differences in PDT efficiency were still observed. We hypothesize that these differences are based on the fact that Ec1 and Ac2 target different epitopes of EpCAM [43]. It could be that more Ec1 DARPins are able to bind EpCAM due to a more accessible epitope. A second explanation might be that Ac2 binds EpCAM at a different epitope and might position itself further away from the plasma membrane than Ec1. The ROS that are produced with PDT are only very short-lived and therefore only act at short distances [12]. Alternatively, a third explanation is that Ac2 has a much faster off-rate compared to Ec1 [43]. This will decrease the bound levels of Ac2 in the time needed for washing between incubation and illumination. A fast off-rate might also explain the observation that incubation with Off7, despite showing some non-specific binding, did not result in cell killing.

Various mechanisms for induction of cell death by IRDye 700DX-conjugates have been proposed. There is evidence for involvement of ROS-mediated cytotoxicity, since therapeutic effects could partly be blocked with scavengers of ROS [47,48,49]. More recently, an alternative mechanism entailing photoinduced physicochemical changes of IRDye 700DX and subsequent loss of cell membrane integrity was described [50,51]. Here, we confirmed that singlet oxygen generation was similar for DARPin-IRDye 700DX as compared to the free IRDye 700DX, although we did not observe a reduction in phototoxicity of 50 nM Ec1-IRDye 700DX in the presence of ROS scavengers. Given that the phototoxicity was very potent at 50 nM, our findings do not rule out an involvement of ROS and are compatible with contributions to phototoxicity of both proposed mechanisms.

In subsequent experiments, distribution of the DARPin conjugates and tPDT efficacy in 3D models were analyzed. Spheroids showed an increased signal at the rim of the spheroid for Ec1, which rapidly decreased when moving further into the spheroid, indicative of a binding site barrier. This phenomenon has been described before for high-affinity antibodies and antibody fragments [52,53]. For Ac2, the lower affinity for EpCAM may have caused the DARPin to diffuse more rapidly and homogenously into the spheroid, presumably because of the higher off rate, thereby avoiding the frozen front that is typical of a binding site barrier. Despite the homogenous distribution, intensity levels were lower for Ac2, especially at early time points. This may also explain the lack of a positive signal after 4 h of binding to primary tissue (Figure 6B). The absence of penetration for Off7 demonstrates that penetration is in any case a function of binding as also observed for elastin-like peptide nanoparticles decorated with a cell-penetrating peptide [44].

A high degree of specificity is of crucial importance to overcome side effects that are currently observed in the clinic with non-targeted photodynamic therapy. Specificity is primarily obtained by binding to cancerous cells expressing high levels of accessible EpCAM. We showed that Ec1-IRDye 700DX and Ac2-IRDye 700DX bound only to EpCAM-expressing cells, and this translated to a lack of cytotoxicity of EpCAM-negative E98 cells. Furthermore, specificity of DARPin-IRDye 700DX conjugates was shown in a co-culture experiment with OVCAR-3 cells and C5120 fibroblasts. This experiment showed that the bystander effect was minimal and that only the tumor cells were affected by PDT. Since cocultures are only a very simplified model of the tumor environment, we also assessed tumor cell specificity in primary tumor material in two different ways. In frozen tissue sections derived from human ovarian carcinoma, both Ec1 and Ac2 bound specifically to tumor cells and not to other cell types. This specificity was confirmed in the tumor explants model in which samples were exposed to the DARPins before fixation.

During targeted photodynamic therapy, dual-specificity is obtained by activation of the photosensitizer bound to the target cells with light which has limited penetration depth in biological tissues [54]. In case of metastasized ovarian cancer, light can specifically be applied onto the parietal and visceral lining of the peritoneal cavity after surgical tumor debulking, as has been done before in the clinical trials for non-targeted photodynamic therapy [15,16]. In this way, other structures that are known to express EpCAM, such as the epithelial lining of the colon and the fallopian tubes, can be protected.

Preclinical models for disseminated ovarian cancer are available; however, we chose to perform our pilot in vivo studies in mice carrying subcutaneous OV90 xenografts because of the homogenous tumor growth, ease of measuring tumor size and standardized histological work-up. In ex vivo fluorescence imaging studies, high accumulation of the DARPin-conjugates in the kidneys of these mice was observed, in analogy to what is described in the literature [55]. This kidney enrichment can be attributed to the small size of the DARPins, which permits filtration through the glomerular filtration barrier. Nevertheless, we observed accumulation of Ac2- and Ec1-IRDye 700DX in the tumors and this accumulation was EpCAM specific as no enrichment was present for Off7. Interestingly, in a recent report on radiolabeled Ec1, a higher relative tumor accumulation as compared to the kidney was observed [56], which was attributed to the non-residualizing nature of the radiolabel [57]. Assuming that the IRDye 700DX remains intracellularly, high accumulation of IRDye 700DX-fluorescence in non-target organs such as the kidney can be explained by normal mechanisms of clearance of the DARPins.

In our study, no phototoxicity was observed in the tumor tissue upon illumination. The experimental set-up and various factors such as tumor size, light dose, protein dose and time after illumination to analyze tissue damage were based on literature reports and successful tPDT experiments with other tracers in our laboratory [58,59,60]. One major difference is that many of these experiments use cells with artificial overexpression of the target or cell lines with endogenously extremely high target expression, such as the A431 cell line for the epidermal growth factor receptor [47]. Furthermore, some papers report on illuminating the tumor twice, on the day of injection and the day after [61,62,63]. Although these models can be used to deliver a proof of concept, we believe that using cells with physiological expression of the target will provide more useful information for clinical translation. Furthermore, illuminating once will resemble the clinical situation better. In the case of metastasized ovarian cancer, which is the long-term perspective for our studies, illumination will be performed once during surgery, after tumor debulking.

Given the rapid systemic clearance of DARPins, which is a downside of the small molecular weight, in future experiments, the effect of half-life extending moieties, such as PEG or an albumin-binding domain [64,65], should be explored for prolonging the plasma half-life, potentially leading to a higher tumor accumulation and greater efficacy. These agents would still be significantly smaller than IgG antibodies and also could be engineered to have a local triggered release of the targeted photosensitizer. Ultimately, a balance needs to be found between a long plasma half-life, which by itself is undesirable, and the need for sufficient tumor accumulation.

## 4. Materials and Methods 

### 4.1. Recombinant DARPin Production

Reading frames for anti-EpCAM DARPins Ec1 and Ac2, and the anti-maltose-binding protein DARPin Off7 [66] were cloned as gBlocks (Integrated DNA technologies, Coralville, IA, USA) in the pQIq vector [66], resulting in DARPins with an N-terminal MRGSHHHHHH and a C-terminal GGSGCGGS sequence followed by a FLAG tag to allow Ni-NTA bead-based purification, maleimide-thiol chemistry and detection, respectively. For some experiments, DARPins were recloned into pHEN-IX plasmids, to yield VSV-tagged DARPins [37].

Recombinant proteins were expressed via IPTG induction in the E. coli strain BLR(DE3) and purified using immobilized metal ion affinity chromatography (IMAC) as described before [66]. Purity was confirmed via SDS-PAGE and Bio-Rad stain-free imaging technology or Coomassie blue-staining.

### 4.2. DARPin Conjugation

Production of DARPin-PEG4-DBCO and DARPin-fluorophore conjugates: DARPins (100–200 µM) were reduced with 100× molar excess of tris(2-carboxyethyl)phosphine (TCEP) in Tris-buffered saline (TBS; 20 mM Tris-HCl, 150 mM NaCl, pH 7.4) for 30 min at 37 °C. After removal of TCEP by buffer exchange on 7 kDa Mw cutoff ZEBA spin columns (Thermo Fisher Scientific) in degassed 4-(2-hydroxyethyl)-1-piperazineethanesulfonic acid (HEPES) buffer (50 mM HEPES, 150 mM NaCl, pH 7.2) a 2× molar excess of fluorescein-5-maleimide (Thermo Fisher Scientific), Alexa Fluor 680 C2 maleimide (Thermo Fisher Scientific, Waltham, MA, USA) or a 4× molar excess of dibenzocyclooctyne (DBCO)-polyethylene glycol (PEG)4-maleimide (Jena Bioscience, Jena, Germany) was added. The reaction was allowed to proceed for 2 h at room temperature before quenching with a 10× molar excess of dithiothreitol (DTT). DARPin conjugates were dialyzed with a 3.5 kDa membrane (Spectra/Por) against PBS overnight at 4 °C and stored at −80 °C until use. 

Production of IRDye 700DX-N_3_: IRDye 700DX-NHS (Li-Cor, Bad Homburg, Germany) was equipped with an azide group by reacting it with NH_2_-PEG3-N_3_ (Jena Bioscience) and triethanolamine (Sigma Aldrich, St. Louis, MO, USA) overnight in anhydrous DMF at 30 °C at molar ratios of 1:1.5:2.25, respectively. The solution was then lyophilized and rehydrated in PBS. 

Production of DARPin-PS conjugates: DARPin-PEG4-DBCO was mixed with IRDye 700DX-N_3_ at a 1:2 molar ratio (DARPin:IRDye 700DX) and incubated overnight at 4 °C followed by extensive dialysis to remove unreacted IRDye 700DX conjugate.

DARPin-conjugate concentrations were assessed by ultraviolet-visible spectroscopy (UV-VIS) on a NanoDrop 2000c (Thermo Fisher Scientific). For fluorescein conjugates, the concentration was determined using the ε at 280 nm of the protein while correcting for the absorption of fluorescein at 280 nm (0.3 × absorption at 495 nm). The protein concentration was determined by measuring absorbance at 679 nm for Alexa Fluor 680 conjugates (ε = 183,000 M^−1^cm^−1^) and at 689 nm for IRDye 700DX conjugates (ε = 165,000 M^−1^cm^−1^) using the Nanodrop spectrophotometer. Complete 1:1 labeling was assumed.

### 4.3. Cell Culture and Spheroid Production

Ovarian cancer cell lines OVCAR-3 (HTB-161, ATCC, Manassas, VA, USA), SKOV-3 (HTB-77, ATCC) and OV90 (CRL-11732, ATCC), astrocytoma cell line E98 [67] and primary skin fibroblasts C5120 [46] were cultured in a humidified incubator at 37 °C and 5% CO_2_. SKOV-3 and E98 cells were cultured in DMEM (Gibco) with 10% fetal calf serum (FCS). OVCAR-3 cells were cultured in RPMI (Gibco) with 20% FCS. Following recommendation by ATCC, OV90 cells were cultured in MCDB 105 (Cell Applications) and Medium 199 (Sigma-Aldrich) (1:1, v:v) with 15% FCS. C5120 cells were cultured in Medium 199 with 10% FCS. 

Tumor cell spheroids of OV90 and SKOV-3 cells were formed by the hanging drop method as described earlier [68]. Cells were detached with trypsin/EDTA and resuspended in complete culture medium containing 1.2 mg/mL methylcellulose (Sigma-Aldrich) with 200 U/mL penicillin/streptomycin (Sigma-Aldrich). Then, 15,000 cells were seeded in 30 µL drops hanging from the inverted lid of a 140 × 20.6 mm petri dish (VWR international). Spheroids were used for experiments after 48 or 72 h. 

### 4.4. Flow Cytometry

Cells were detached with 5 mM EDTA in PBS and 1.0 × 10^5^ cells per condition were seeded into a V-bottom 96-well plate (Sigma-Aldrich). DARPin-fluorescein and antibodies were incubated for 1 h in PBS containing 3% (w/v) bovine serum albumin (BSA) at 4 °C. Mouse-anti-EpCAM (VU-1D9, Invitrogen, Waltham, MA, USA) and goat-anti-mouse Alexa Fluor 488 (A-11029, Thermo Fisher Scientific) were incubated at 5 µg/mL. The samples were washed three times between each incubation and measured on a FACSCalibur flow cytometer (BD Bioscience, Erembodegem, Belgium). Flow cytometry data were analyzed with Kaluza software (Beckman Coulter, Brea, CA, USA).

### 4.5. Confocal Microscopy 

First, 2.0 × 10^4^ cells were seeded in each well of 8-well μ-slides (80,826, Ibidi). After overnight incubation, the samples were treated with 200 nM DARPin-fluorescein for 30 min at 37 °C in 100 μL complete culture medium. Cells were washed three times with complete culture medium and imaged in complete phenol-red free culture medium with 10 mM HEPES. Confocal microscopy was performed on a TCS SP5 confocal microscope (Leica Microsystems, Wetzlar, Germany) with an HCX 63 × 1.20 N.A. water lens. Fluorescein was excited with the 488 nm line of an argon ion laser and emission was recorded with a PMT detector between 500 and 550 nm.

Spheroids were incubated with 500 nM DARPin-Alexa Fluor 680 conjugates in hanging drops. After 30 min and 2.5 h, spheroids were rinsed once, washed twice for 10 min with complete culture medium and once with PBS, before fixating them in 4% paraformaldehyde (PFA) for 1–2 h at room temperature. After fixation, PFA was diluted to 2% with PBS and stored at 4 °C until collagen embedding for clearing as described before [69].

To clear spheroids, a previously described adaptation [69] of the SeeDB protocol [70] was employed. Both protocols use increasingly concentrated fructose solutions supplemented with 0.5 % (v/v) α-thioglycerol. After clearing, the spheroids were imaged with the SP8 SMD confocal microscope (Leica Microsystems) to visualize penetration of DARPin-Alexa Fluor 680 conjugates. The Leica 10 × 0.4 N.A. dry lens was used and Alexa Fluor 680 was excited with the 670 nm laser line of the white light laser and emission was detected with a HyD detector from 750 to 790 nm. Quantification of penetration depth of DARPin-Alexa Fluor 680 conjugates into spheroids was performed as described earlier [44]. Quantitative analyses of spheroid uptake were performed with Fiji ImageJ software [71], as described before [44].

### 4.6. Singlet Oxygen Generation by DARPin-IRDye 700DX Conjugates

Generation of singlet oxygen by IRDye 700DX or DARPin-IRDye 700DX conjugates upon illumination was determined by measuring reduction of absorbance of two singlet oxygen reporter molecules p-nitrosodimethylaniline (RNO) or 9,10-anthracenediylbis(methylene) malonic acid (ABDA). 500 nM of the DARPin-IRDye 700DX conjugates were incubated with either 50 µm RNO (Sigma-Aldrich) and 400 µM imidazole (Sigma-Aldrich) or with 185 µM ABDA (Sigma-Aldrich) in 20 mM sodium phosphate buffer, pH 7.4. Absorbance at either 440 nm for RNO or at 378 nm for ABDA was measured (CLARIOstar plate reader, BMG Labtech, Ortenberg, Germany) after various periods of illumination with 100 mW/cm^2^ 690 nm light. 

### 4.7. tPDT of Adherent Cell Cultures and Spheroids

All experiments were performed in triplicate. One day prior to an experiment, 1.5 or 2.0 × 10^4^ cells were seeded per condition in complete culture medium in 96-well plates. The cells were incubated for 30 min with serial dilutions of DARPin-IRDye 700DX constructs (ranging from 0.5 to 500 nM or as indicated), washed 3× and illuminated with a custom-built 690 nm LED for 10 min at 100 mW/cm^2^ (total light dose of 60 J/cm^2^) and cultured further. While varying the total light dose (30–90 J/cm^2^), the dose rate was kept constant (100 mW/cm^2^). While varying the dose rate (50–200 mW/cm^2^), the total light dose was kept constant (60 J/cm^2^). The ROS scavengers L-histidine and sodium azide were incubated in complete medium at indicated concentrations for 1 h with OVCAR-3 cells after a 30 min incubation with 50 nM Ec1-IRDye 700DX. The scavengers were washed away after the illumination. The next day, cells were incubated with 100 µL 0.1 mg/mL resazurin (Sigma-Aldrich) in complete culture medium at 37 °C to quantify surviving cells. After 2 h, fluorescence was measured with a BioTek Synergy 2 Multi-Mode Reader (BioTek Instruments, Winooski, VT, USA). Excitation was 540/25 nm and emission was 620/40 nm. To correct for fluctuations in cell density between independent experiments, cell viability was normalized to the 0.5 nM sample. 

For tPDT of spheroids, seven spheroids per condition were incubated with 500 nM DARPin-IRDye 700DX conjugates. After 2 h, spheroids were transferred to a 1.5% (w/v) agarose-coated 12-well plate. Spheroids were rinsed briefly and washed twice for 30 min with complete culture medium. Each spheroid was transferred to an individual well of a 96-well agarose-coated plate. The samples were illuminated with a 690 nm LED for 10 min (100 mW/cm^2^) and incubated overnight.

To determine viability of spheroids, the acid phosphatase (APH) assay was performed as described before with adjustments [45]. In short, spheroids were washed twice by centrifugation for 10 min at 600× *g*. Samples were incubated with an equal amount of APH buffer (0.1 M sodium acetate, 0.1% (v/v) Triton-X-100 and 2 mg/mL p-nitrophenyl phosphate solution in milliQ water) for 90 min at 37 °C. After incubation, 10 µL of 1 M NaOH was added to deprotonate the reaction product and absorption was measured at 405 nm within 10 min. Normalized cell viability was calculated with the non-treated and non-illuminated spheroids set as 100%.

### 4.8. PI-Calcein AM Stain after tPDT

First, 4.5 × 10^4^ OVCAR-3 cells per well were seeded in an 8-well μ-slide. The next day, samples were incubated with 60 or 15 nM DARPin-IRDye 700DX conjugates for 30 min. Afterwards, the wells were washed three times with medium and illuminated with a 690 nm LED for 10 min at 100 mW/cm^2^. Hereafter, the cells were incubated for 15 min with 10 µg/mL propidium iodide (PI) and 4 µM calcein AM before imaging on the TCS SP5 confocal microscope with the 63 × 1.20 N.A. water lens.

Image analysis was performed with Fiji ImageJ software. To calculate the percentage of dead cells in each sample, three images per sample were acquired and divided into four quadrants. Uneven illumination in the calcein channel was corrected with an FFT bandpass filter, followed by thresholding of the calcein and PI channels to remove background signal, dilation of particles and a watershed operation, after which particles in the calcein and PI channels were counted. To correct for double positive cells, the calcein mask was subtracted from the PI mask and remaining particles were counted, representing PI-positive/calcein negative cells. The difference between the total PI-positive cell count and the single PI-positive cell count represented the double positive cells. To calculate the number of live cells (calcein positive/PI negative), the double positive cell count was subtracted from the calcein positive cell count. The percentage of dead cells was calculated by dividing the number of dead cells (total PI-positive cell count) by the total number of cells. 

### 4.9. tPDT of Co-Cultures in Matrigel

OVCAR-3 cells and C5120 fibroblasts were stained with 5 µM carboxyfluorescein succinimidyl ester (CFSE, Invitrogen) or 5 µM CellTrace Red (Invitrogen), respectively. The cells were co-cultured in a ratio of 1:1 in phenol red-free Matrigel (Corning, New York, NY, USA) in a 96-well plate. Matrigel was allowed to solidify at 37 °C for 30 min. After solidification, 100 µL medium (complete M199) was added to each well and the plate was incubated overnight at 37 °C.

The following day, co-cultures were incubated with 20 or 100 nM of Ec1 or Off7 DARPin-IRDye 700DX conjugates for 2 h. The samples were rinsed and washed twice for 15 min. The plate was illuminated with a 690 nm LED for 10 min at 100 mW/cm^2^. After illumination, 100 µg/mL PI was added and incubated for 45–60 min to stain late apoptotic and necrotic cells. Confocal microscopy was used to visualize CFSE-stained (OVCAR-3), CellTrace Red-positive (C5120 fibroblasts) and PI-positive cells. The cells were imaged with confocal microscopy (TCS SP5) by making z-stacks of 245 µm total thickness (one slice per 10 µm).

To quantify the number of viable and late dead cells, a threshold was applied to all slices in the z-stack to remove background signal in each channel separately. Particles were dilated and a watershed was applied. The number of particles was counted for all individual channels using the 3D Objects Counter plugin in Fiji. The number of dead OVCAR-3 cells was calculated by subtracting the mask of CellTrace Red (C5120 cells) from the mask of PI, and then subtracting the remaining PI-positive structures from the total of PI-positive structures. The number of dead C5120 cells was calculated by subtracting the mask of CFSE (OVCAR-3 cells) from the mask of PI and then subtracting the remaining PI-positive structures from the total of PI-positive structures. A correction for PI-positive and CFSE- and CellTrace Red-negative structures was taken into account.

### 4.10. OV90 Xenograft Animal Model

Tumor cell injection: 1–3 million OV-90 cells in 100 µL serum-free culture medium with Matrigel (1:1) were injected subcutaneously on both the left and right upper hind paw of 6–8 weeks old female BALB/c nude mice (Janvier, Le Genest Saint Isle, France).

Fluorescence imaging: In a pilot experiment, when tumors reached sizes of 100 mm^3^, mice were injected intravenously with 2 (*n* = 1) or 4 nmol (*n* = 1) of the IRDye 700DX conjugated DARPins in 100 µL PBS. After 4 h, mice were sacrificed by CO_2_ asphyxiation, and their organs were dissected and fluorescence imaging was performed using the IVIS lumina closed-cabinet fluorescence scanner (Caliper LifeSciences, Hopkinton, MA, USA) (excitation 640 nm; background correction 535 nm). After scanning, organs were snap frozen in OCT tissue tek and cut into 4 µm sections. Sections were mounted with fluoromount containing DAPI (Thermo-Fisher Scientific) and a coverglass and imaged with the EVOS FL imaging system with LED light cube Cy5 (Thermo Fisher Scientific).

In Vivo photodynamic therapy: Mice were randomized (*n* = 8 per group) and injected intravenously with 4 nmol of the IRDye 700DX-conjugated DARPin constructs in 100 µL PBS, or PBS only as control. After circulation for 4 h, mice were anesthetized using isoflurane, and their bodies except for one of the two tumors were shielded from light with wet tissues and aluminum foil. The exposed tumor was illuminated for 14 min and 42 s with 170 mW/cm^2^ 690 nm light, reaching a total light dose of 150 J/cm^2^ using a dedicated LED device. Twenty-four hours after illumination, mice were sacrificed by CO_2_ asphyxiation. Tumors were harvested, fixed overnight in 4% buffered formalin, and paraffin embedded. Four-micrometer sections were used for immunohistochemistry.

### 4.11. HE and Immunohistochemistry for Cleaved Caspase-3 and γH2A.X

Tissue sections were stained with hematoxylin and eosin to visualize morphology. Furthermore, sections were stained for the apoptosis markers cleaved caspase-3 and DNA double stranded break marker γH2A.X using immunohistochemistry [72]. In short, sections were deparaffinized with xylene and rehydrated in ethanol. Antigen retrieval was performed for 10 min at 96 °C in 10 mM citrate pH 6.0 in a PT-module (Thermo Fisher Scientific). For cleaved caspase-3 stainings, endogenous peroxidases were quenched with 3% H_2_O_2_ in PBS for 20 min at room temperature. Then, slides were incubated with 20% normal goat serum and 1% BSA in PBS for 30 min at room temperature, and subsequently with 1:4000 rabbit-anti-cleaved caspase 3 (Cell Signaling Technology, Danvers, MA, USA) in PBS (1% BSA) in a humidified chamber overnight at 4 °C. For γH2A.X stainings, slides were first blocked with 20% normal goat serum and 1% BSA in PBS for 30 min at room temperature. Subsequently, slides were incubated overnight in a humidified chamber at 4 °C with 1:2000 rabbit-anti- γH2A.X (Cell Signaling Technology), and after that endogenous peroxidates were blocked by incubation in 0.3% H_2_O_2_ in methanol for 20 min at room temperature. Subsequently, for both stainings, slides were incubated with 1:200 goat-anti-rabbit-biotin (Vector laboratories, Peterborough, UK) in PBS (1% BSA) for 30 min at room temperature, and avidin-biotin complex (ABC, Vector laboratories) in PBS (1% BSA) for 30 min at room temperature. Bound antibodies were visualized using bright 3,3′-Diaminobenzidine (DAB; ImmunoLogic, Duiven, NL, USA) for 8 min at room temperature. Counterstaining was performed with hematoxylin for 10 s, and slides were mounted with permount (Thermo Fisher Scientitic) and a cover slip.

### 4.12. Immunohistochemistry of Frozen Tissue Sections 

Fast-frozen tissue of papillary serous ovarium carcinoma was stored under the standard regulations of the Radboud University Medical Center and collected from the Radboud Biobank. Sections of 4 µm were sliced with a cryostat, fixed for 10 min in ice-cold acetone and air dried. The cryosections were subsequently incubated for 1 h with 250 nM DARPin in Normal Antibody Diluent (ImmunoLogic) followed by 1:200 mouse anti-VSV in Normal Antibody Diluent for 1 h, and finally by Powervision with Poly-HRP-Anti-Mouse/Rabbit/Rat for 30 min. The anti-VSV antibody (clone P5D4) was a kind gift of Toin van Kuppevelt (Dept. of Biochemistry, Radboud University Medical Center, Nijmegen, The Netherlands). Controls were incubated with 1:200 Mouse-anti-EpCAM (Abcam ab7504) in Normal Antibody Diluent for 1 h followed by Powervision for 30 min. Antibodies were visualized by a 7-min incubation with DAB and a nuclear stain was performed by hematoxylin incubation for 1 min followed by a 5 min rinse with tap water. The slides were dehydrated with ethanol and xylene and mounted with Quick D mounting medium (Klinipath, Duiven, The Netherlands).

### 4.13. DARPin Penetration in Explants 

Tumor explants were cultured as described before [73]. Fresh ovarian tumor metastases were obtained from patients that underwent cytoreductive surgery for stage IIIC high-grade serous adenocarcinoma at the Canisius Wilhelmina Hospital, Nijmegen, The Netherlands.

First, 200 nM DARPin-IRDye 700DX and DARPin-fluorescein conjugates were incubated for 4 and 18 h. They were subsequently rinsed once and washed twice with complete culture medium. The samples that were incubated with fluorescein-conjugates were subsequently fixed in 4% PFA and stored at 4 °C and processed for paraffin embedding. The paraffin embedded blocks were further processed for multiplex immunohistochemistry.

### 4.14. Multiplex Immunohistochemistry

The formalin-fixed tumor slices were paraffin-embedded and sections of 5 µm were cut from the center of a slice. The sections were subjected to four-color multiplex immunohistochemistry in sequential staining cycles using Opal 7-color Automation IHC Kit (NEL801001KT; PerkinElmer) on the BOND RX IHC & ISH Research Platform (Leica Biosystems, Wetzlar, Germany). A multiplex panel was applied on tissue sections consisting of anti-FLAG-tag 1:5000 (A00187-100, clone 5A8E5; GenScript) with Opal 690, anti-EpCAM 1:1000 (ab187372, clone VU-1D9; Abcam) with Opal 570 and anti-pan Cytokeratin 1:1500 (ab86734, clone AE1/AE3 + 5D3; Abcam) with Opal 650. Primary antibody incubations were performed for 1 h, secondary antibody Opal Polymer HRP Ms + Rb incubations for 30 min and Opal reagent incubations for 10 min, all at room temperature. All epitope retrievals and antibody-TSA complex removals were performed using Bond Epitope Retrieval 2 (AR9640, Leica Biosystems, Wetzlar, Germany). Tissue sections were counterstained with DAPI and mounted in Fluoromount-G (0100-01; SouthernBiotech, Birmingham, AL, USA).

The slides were scanned using the Automated Quantitative Pathology Imaging System (Vectra 3.0.4, PerkinElmer, Waltham, MA, USA). Regions of interest were selected using Phenochart (Version 1.0.9, PerkinElmer). InForm software (Version 2.2.1, PerkinElmer) was used for spectral unmixing of Opal fluorophores, DAPI and autofluorescence and downstream image analysis.

Twenty representative multispectral images were used to train the inForm software to distinguish DARPin/FLAG-tag positive tumor regions from negative tumor regions, stroma and background based on DAPI, Opal 650, Opal 690 and autofluorescence signals. All the settings applied to the training images were saved in an algorithm to allow batch analysis of all specimens.

DARPin/FLAG-tag positivity was calculated by dividing the DARPin/FLAG-tag positive tumor surface area by the total tumor surface area (sum of DARPin/FLAG-tag positive and negative tumor surface areas).

### 4.15. Statistical Analyses

All statistical analyses were carried out using GraphPad Prism version 5.03. *P* values < 0.05 were considered significant. For comparisons between two groups, two-tailed unpaired t-tests were performed, assuming normally distributed data. A Bonferroni correction was employed for correcting for multiple comparisons. For comparisons between multiple groups and two independent factors, two-way ANOVAs were performed, using Bonferroni post hoc tests to correct for multiple comparisons.

### 4.16. Ethical Approval

Ethical approval for use of the tumor explants in this study was provided on 23 June 2016 by the Radboud University Medical Center Ethical Committee (file number 2016–2636), which granted the use of peritoneal tumor deposits that were regarded as “leftover material” in accordance with the code of proper secondary use of human tissue in The Netherlands, as established by the Dutch Federation of Medical Scientific Societies.

Ethical approval for the mice experiments in this study was provided on 8 September 2015 by the institutional Animal Welfare Committee of the Radboud University Medical Center (application number AVD103002015209), in accordance with the guidelines of the Revised Dutch Act on Animal Experimentation.

## 5. Conclusions

DARPin-IRDye 700DX conjugates targeted to EpCAM showed high efficacy in 2D monolayers and 3D spheroids and EpCAM-targeting DARPins showed specific binding in primary patient samples. The PDT effect of the DARPin-IRDye 700DX conjugates was restricted to EpCAM-expressing tumor cells. Although Ec1 showed a higher efficacy, Ac2 might still be an interesting candidate because of the fast spheroid penetration. Additional in vivo and ex vivo studies should determine the most promising DARPin and format for further preclinical development.

## Supplementary Materials

The following are available online at https://www.mdpi.com/2072-6694/12/7/1762/s1, Figure S1: IMAC-purified DARPins Ec1, Ac2 and Off7 as assessed by stain-free imaging (Bio-Rad) of an SDS-PAGE gel, Figure S2: Measurement of singlet oxygen generation by reporter molecules, Figure S3: EpCAM expression analyzed by flow cytometry, Figure S4: Binding of DARPin-fluorescein conjugates to EpCAM-expressing ovarian cancer cells, Figure S5: Effect of dose rate and total light dose on phototoxic effects of DARPin-IRDye 700DX conjugates, Figure S6: Effect of ROS scavengers on photoxicity of the DARPin conjugate Ec1-IRDye 700DX, Figure S7: Penetration of DARPin conjugates in OV90 spheroids, Figure S8: Photodynamic therapy of OVCAR-3 cells and C5120 cells seeded in a 3D Matrigel co-culture, Figure S9: Representative images of HE, cleaved caspase-3 and yH2A.X stainings of subcutaneous OV90 tumors.
