# Supplementary material for: EpCAM-Binding DARPins for Targeted Photodynamic Therapy of Ovarian Cancer
Source: Cancers (Basel). 2020 Jul 2;12(7):1762. doi: 10.3390/cancers12071762 (PMC7409335; doi:10.3390/cancers12071762)
Supplement: Supplementary file 1 [file cancers-12-01762-s001.pdf]

cancers

MDPI

Article

EpCAM-Binding DARPins for Targeted Photodynamic Therapy of Ovarian Cancer

Dirk van den Brand, Sanne A.M. van Lith, Jelske M. de Jong, Mark A.J. Gorris, Valentina Palacio-Castañeda, Stijn T. Couwenbergh, Mark R.G. Goldman, Inge Ebisch, Leon F. Massuger, William P.J. Leenders, Roland Brock and Wouter P.R. Verdurmen

Supplementary Materials:

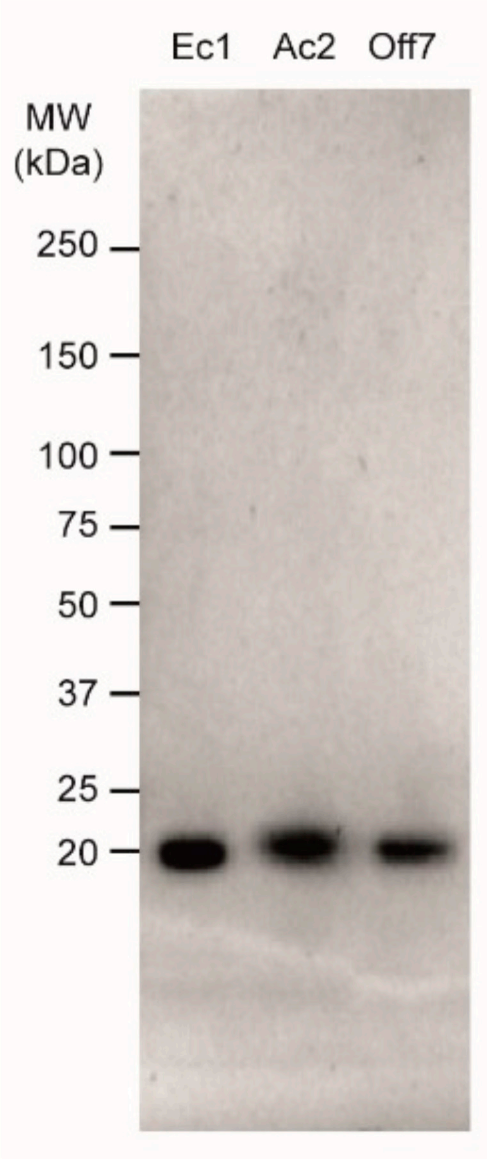

Figure S1. IMAC-purified DARPins Ec1, Ac2 and Off7 as assessed by stain-free imaging (Bio-Rad) of an SDS-PAGE gel.

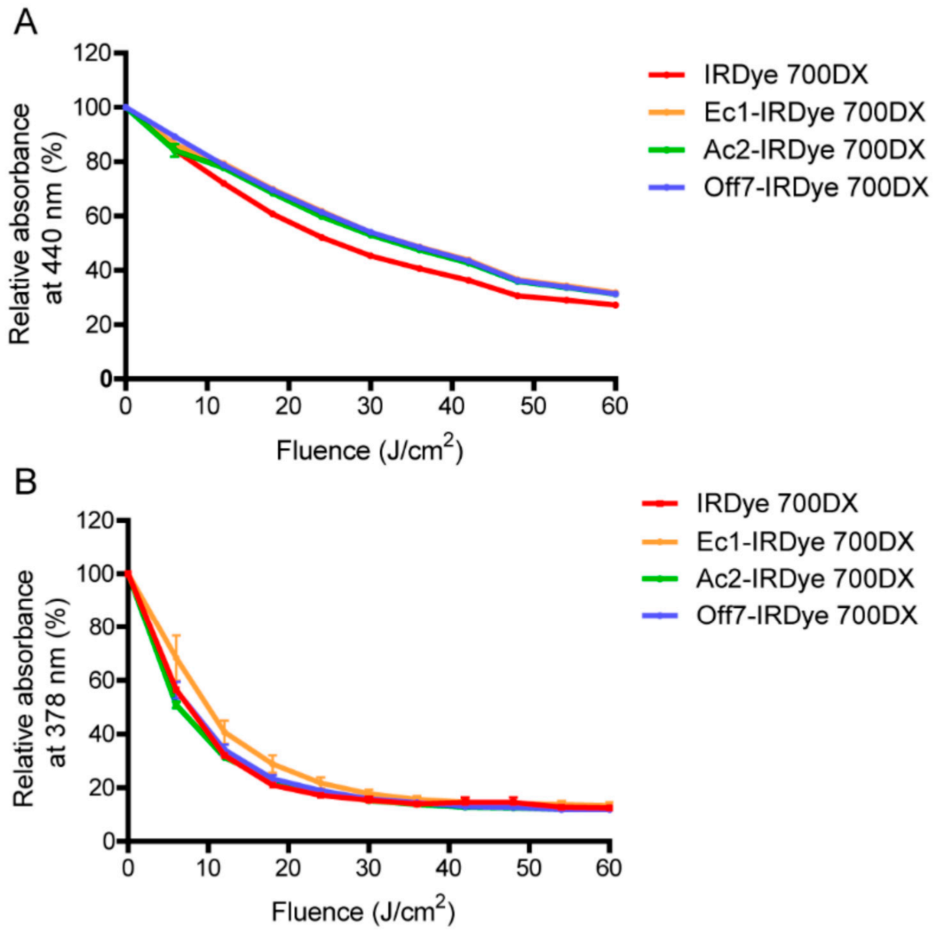

Figure S2. Measurement of singlet oxygen generation by reporter molecules. (A) Bleaching of RNO upon illumination of DARPin-IRDye 700DX conjugates or free IRDye 700DX with near-infrared light (690 nm) as a function of fluence. (B) Bleaching of ABDA upon illumination of DARPin-IRDye 700DX conjugates or free IRDye 700DX with near-infrared light (690 nm) as a function of fluence.

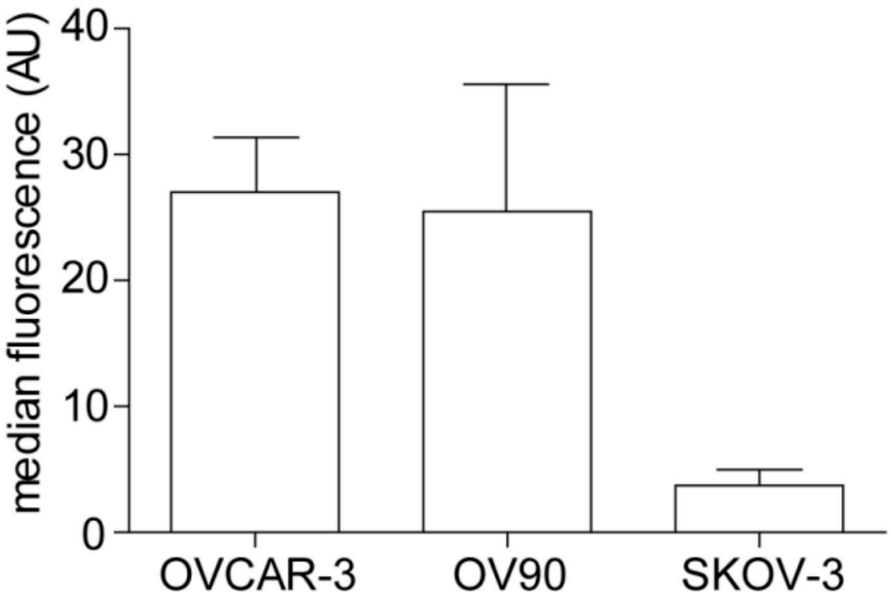

Figure S3. EpCAM expression analyzed by flow cytometry. Cells were incubated with an anti-EpCAM antibody (Abcam ab7504). Error bars reflect s.e.m.; $n = 3$.

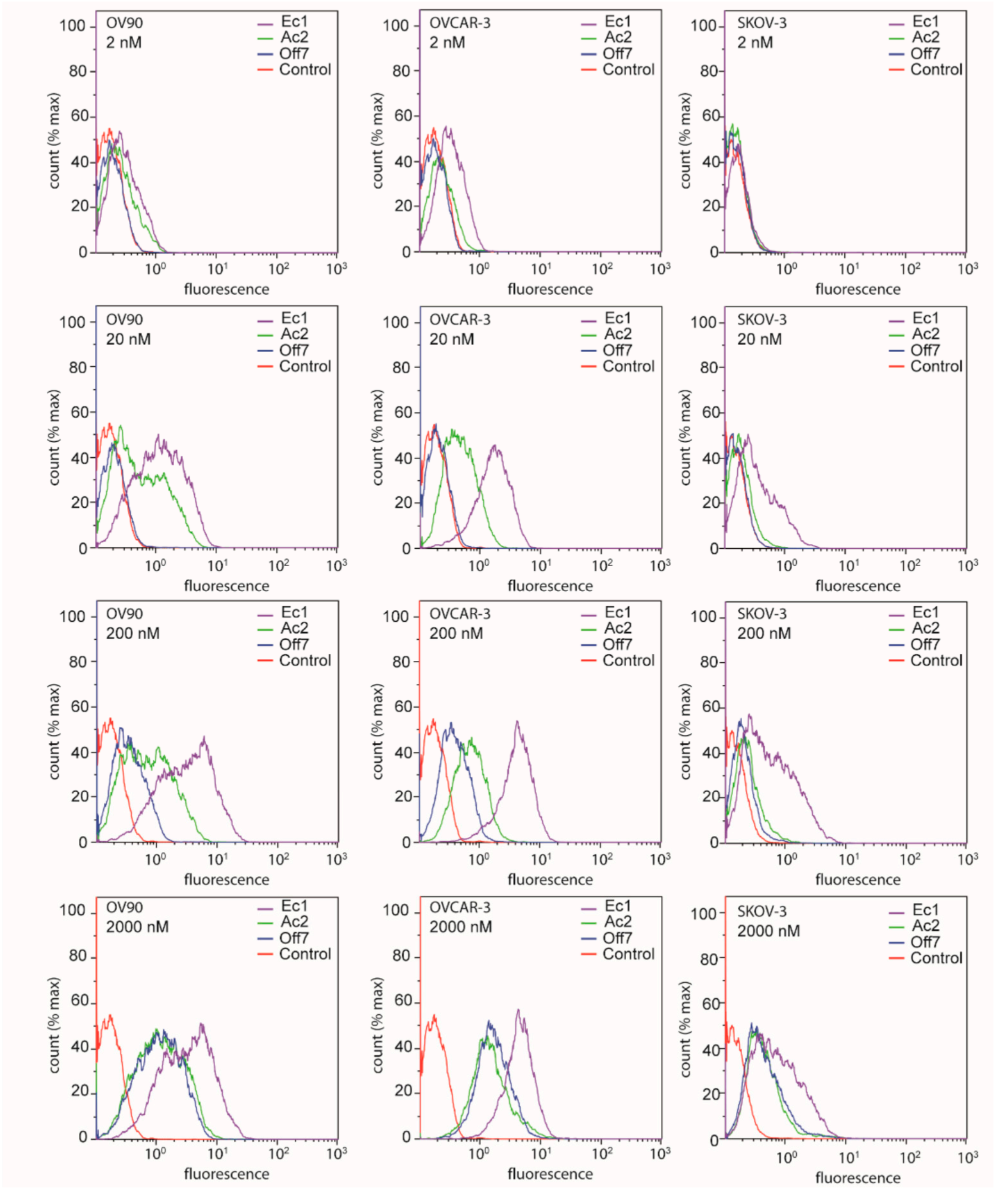

Figure S4. Binding of DARPin-fluorescein conjugates to EpCAM-expressing ovarian cancer cells. Flow cytometry was performed with OV90, OVCAR-3 and SKOV-3 cells incubated with different concentrations of DARPin-fluorescein conjugates. Representative histograms for each condition are depicted.

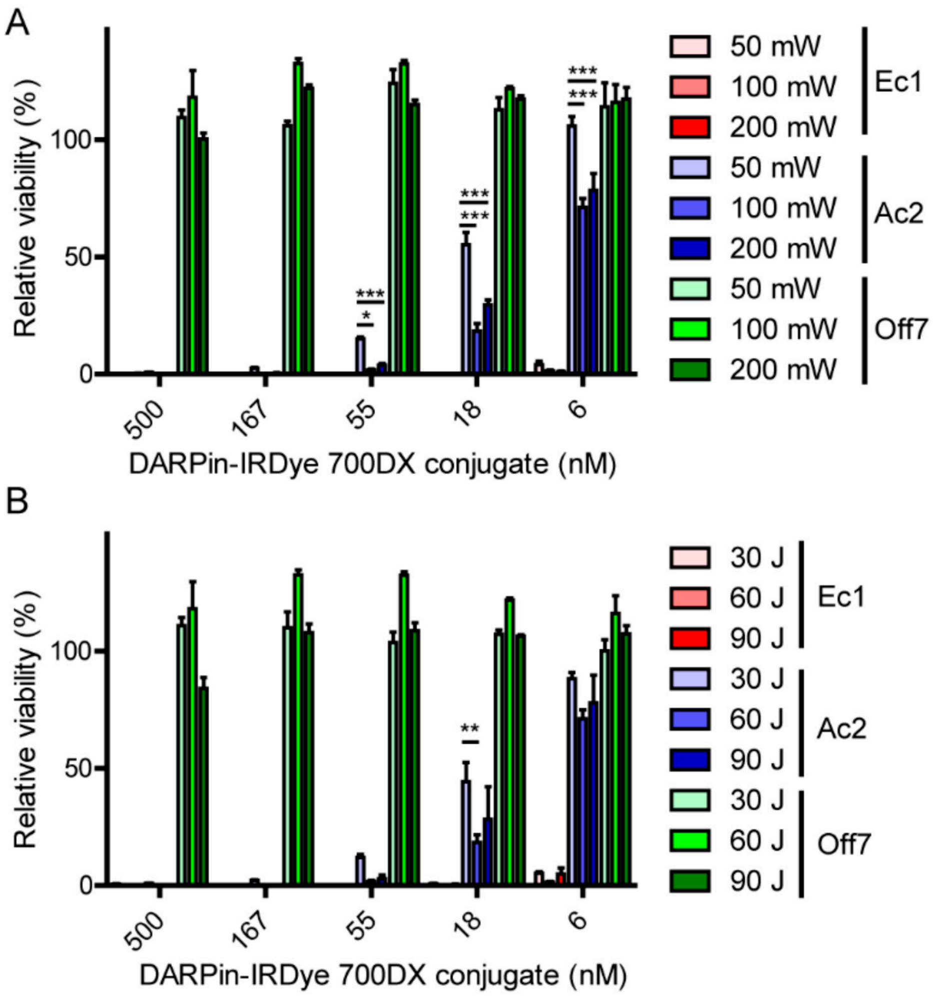

Figure S5. Effect of dose rate and total light dose on phototoxicity of DARPin-IRDye 700DX conjugates. OVCAR-3 cells were incubated with indicated concentrations of DARPin-IRDye 700DX conjugates and illuminated (A) with a varying dose rate and a fixed total dose of 60 J/cm^2 with light of 690 nm or (B) with a fixed dose rate (100 mW/cm^2) and a variable total dose with light of 690 nm. Mean ± s.e.m. is shown, n = 3 (technical replicates). Results were confirmed in an independent experiment. Light dose and dose rates are expressed per cm^2. A two-way ANOVA with Bonferroni post hoc test was used to calculate statistical differences. * $p < 0.05$, ** $p < 0.01$, *** $p < 0.001$.

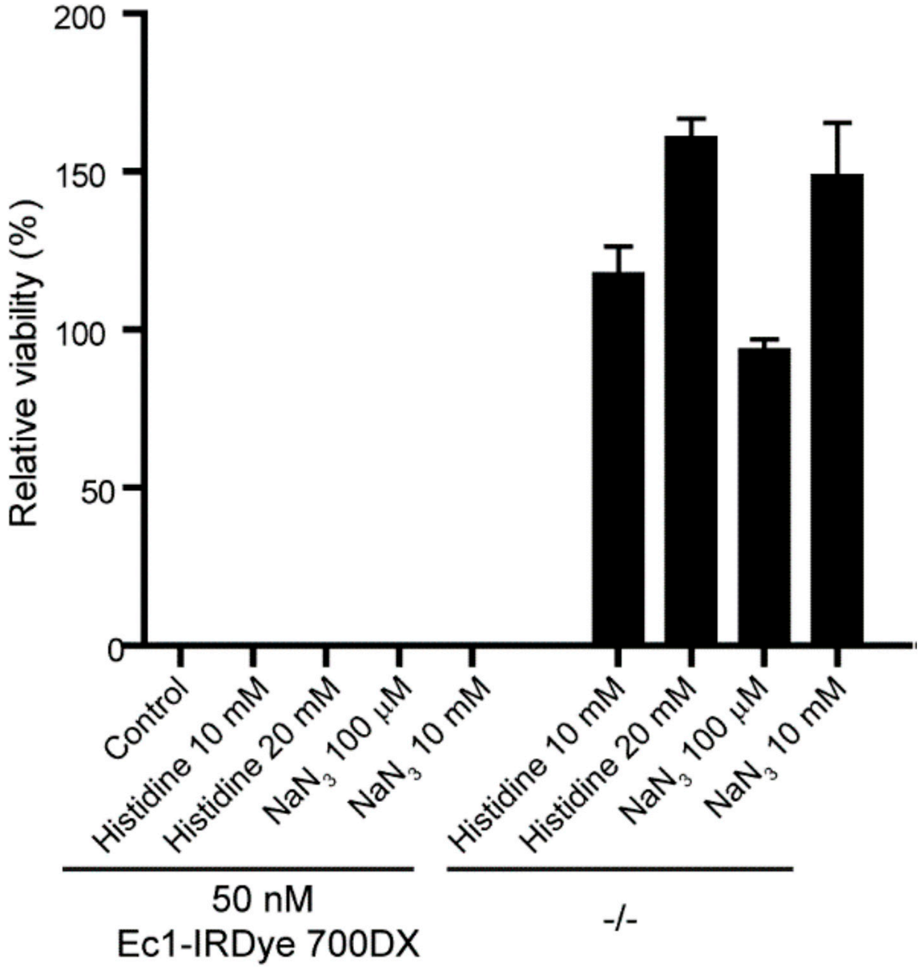

Figure S6. Effect of ROS scavengers on photoxicity of the DARPin conjugate Ec1-IRDye 700DX. OVCAR-3 cells were incubated for 30 min with 50 nM Ec1-IRDye 700DX conjugates, followed by a 1-h treatment with ROS scavengers at indicated concentrations and illuminated with a total light dose of 60 J/cm^2 (100 mW/cm^2) with light of 690 nm. Viability was measured after 24 h with a resazurin assay. Mean ± s.d. is shown, $n = 2$.

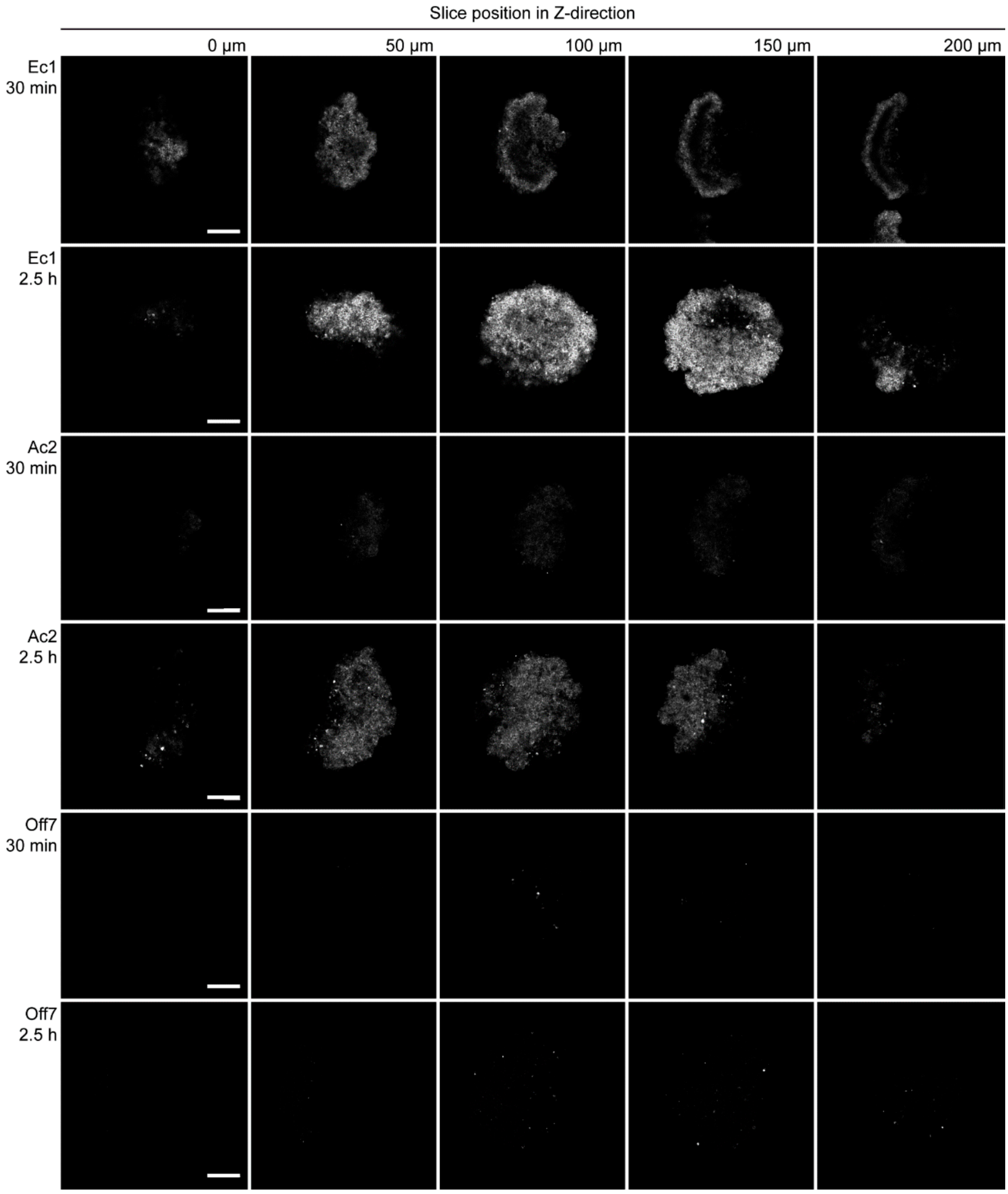

Figure S7. Penetration of DARPin conjugates in OV90 spheroids. OV90 spheroids were incubated with 500 nM DARPin-Alexa Fluor 680 conjugates for the indicated time periods. After the incubation, the spheroids were fixed and cleared. They were subsequently analyzed with confocal microscopy. An image was acquired every 50 µm. The scale bars represent 200 µm.

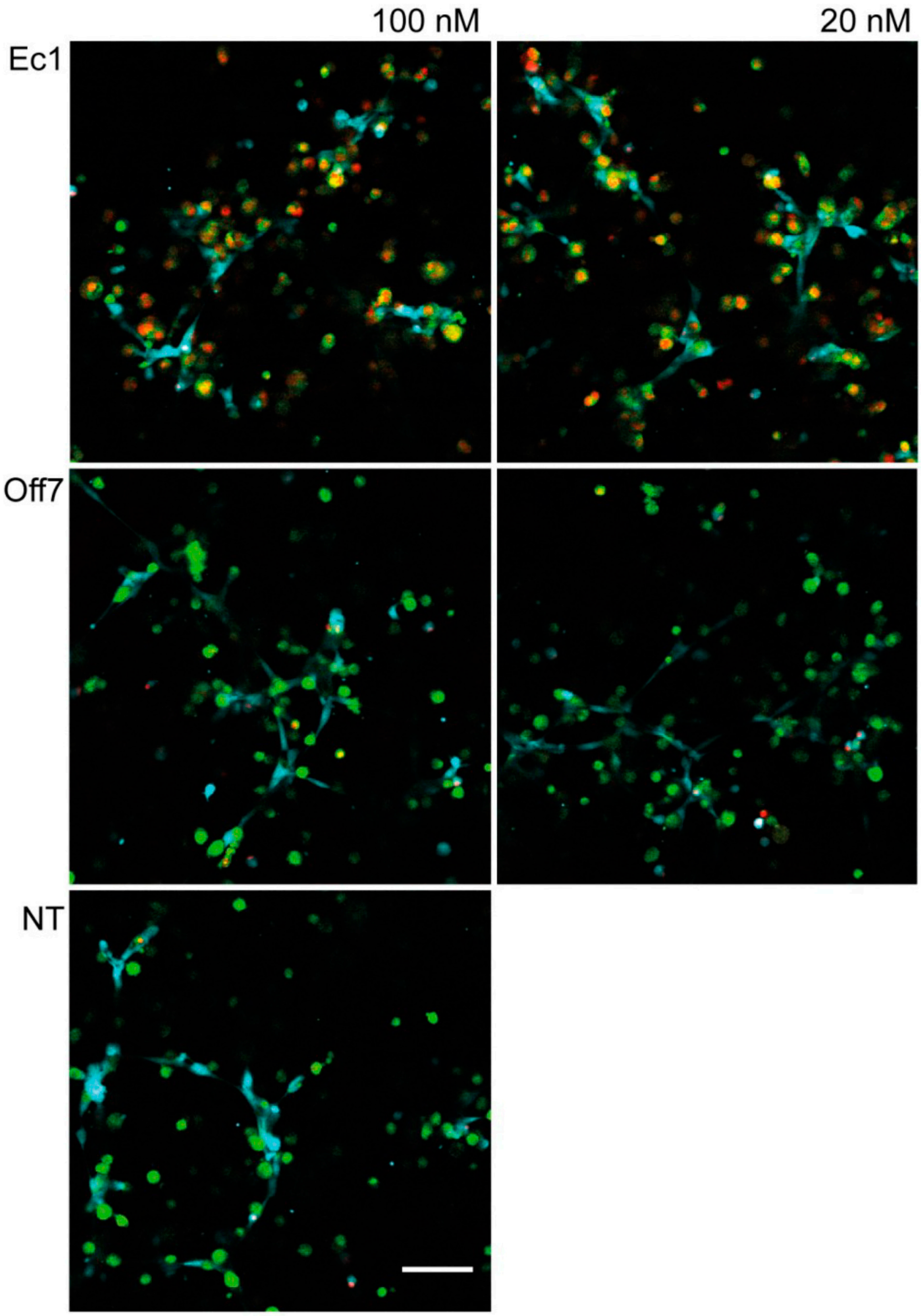

Figure S8. Photodynamic therapy of OVCAR-3 cells (green) and C5120 cells (cyan) seeded in a 3D Matrigel co-culture. Cells were incubated with 100 nM or 20 nM Ec1- or Off7-IRDye 700DX. They were subsequently illuminated with total light dose of 60 J/cm^2. Propidium iodide (red) was added to stain dead cells. The 3D Matrigel cultures were imaged with confocal microscopy. A representative slice of a Z-stack is shown. The scale bar represents 100 μm.

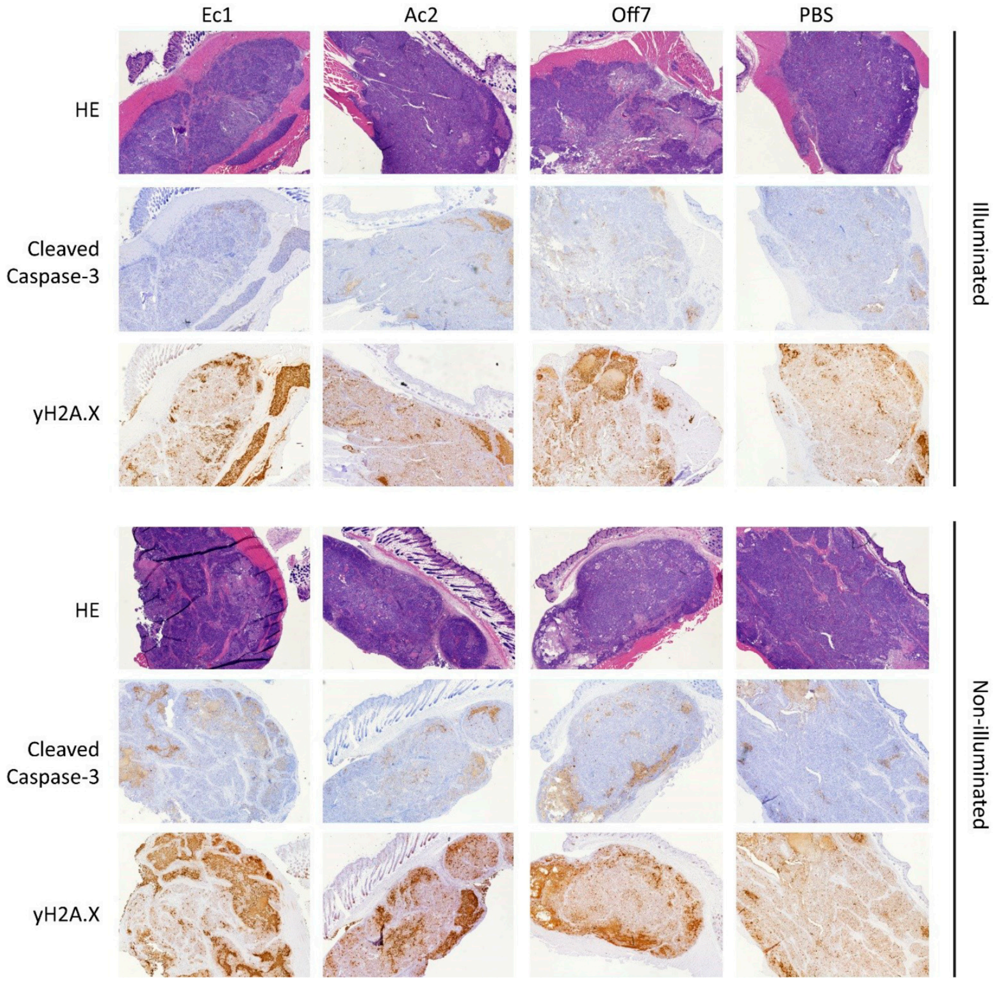

Figure S9. Representative images of HE, cleaved caspase-3 and yH2A.X stainings of subcutaneous OV90 tumors. Mice were injected with Ec1-IRDye 700DX, Ac2-IRDye 700DX, Off7-IRDye 700DX or PBS and tumors were illuminated with 150 J/cm^2 690 nm light. Non-illuminated tumors served as controls. Note that there are no differences in staining intensities, indicating the lack of treatment effect.

© 2020 by the authors. Submitted for possible open access publication under the terms and conditions of the Creative Commons Attribution (CC BY) license (http://creativecommons.org/licenses/by/4.0/).
